# Laser-Assisted Photo-Thermal Reaction for Ultrafast Synthesis of Single-Walled Carbon Nanotube/Copper Nanoparticles Hybrid Films as Flexible Electrodes

**DOI:** 10.3390/nano14171454

**Published:** 2024-09-06

**Authors:** Mi-Jeong Kim, Hee Jin Jeong

**Affiliations:** 1 Nano Hybrid Technology Research Center, Korea Electrotechnology Research Institute (KERI), Changwon 51543, Republic of Korea; jeong0124v@gmail.com; 2 Department of Electro-Functionality Material Engineering, University of Science and Technology (UST), Changwon 51543, Republic of Korea

**Keywords:** single-walled carbon nanotubes, copper nanoparticles, photo-thermal conversion, flexible electrodes

## Abstract

The hybridization of single-walled carbon nanotubes (SWCNTs) and Cu nanoparticles offers a promising strategy for creating highly conductive and mechanically stable fillers for flexible printed electronics. In this study, we report the ultrafast synthesis of SWCNT/Cu hybrid nanostructures and the fabrication of flexible electrodes under ambient conditions through a laser-induced photo-thermal reaction. Thermal energy generated from the nonradiative relaxation of the π-plasmon resonance of SWCNTs was utilized to reduce the Cu-complex (known as a metal–organic decomposition ink) into Cu nanoparticles. We systematically investigated the effects of SWCNT concentration and output laser power on the structural and electrical properties of the SWCNT/Cu hybrid electrodes. The SWCNT/Cu electrodes achieved a minimum electrical resistivity of 46 μohm·cm, comparable to that of the metal-based printed electrodes. Mechanical bending tests demonstrated that the SWCNT/Cu electrodes were highly stable and durable, with no significant deformation observed even after 1000 bending cycles. Additionally, the electrodes showed rapid temperature increases and stable Joule heating performance, reaching temperatures of nearly 80 °C at an applied voltage of less than 3.5 V.

## 1. Introduction

Flexible electronics require the interconnection of conductive materials for various applications, such as sensors, electrodes, and integrated circuits [1,2,3,4]. To achieve effective electrically conductive states in the flexible modes, nanocarbons (NCs), such as carbon nanotubes (CNTs) and graphene, have been extensively studied [5,6,7,8,9,10,11]. However, the lower electrical conductivity of NCs compared to commercially available metals presents a challenge for practical device applications. This issue can be addressed by hybridizing NCs with metal nanostructures, where the metals act primarily as conductive fillers and the NCs serve as flexible interconnects. This hybrid approach results in high electrical conductivity even under shape-deformation conditions.

Consequently, flexible electrodes have been developed using NCs hybrid materials combined with noble metal nanostructures, such as gold and silver, due to their high electrical conductivities, low melting points, and high environmental stabilities [12,13,14,15,16,17,18,19]. Nonetheless, the high cost of noble metals limits their commercial application. Recently, there has been considerable interest in hybrid electrodes based on Cu nanostructures due to their cost-effectiveness and relatively high electrical conductivity compared to noble metals [20,21]. In the fabrication of flexible electrodes, hybrid material-based inks or pastes were initially deposited using conventional coating or printing methods, followed by high-temperature thermal treatment for curing and metal sintering [22,23,24]. During thermal treatment, it was crucial to create a vacuum or introduce an inert gas to prevent the oxidation of Cu nanostructures, which could lead to increased processing costs.

Unlike Cu nanoparticle inks, solution-based Cu-complex inks have recently been used for Cu-based printed electrodes due to their cost-effectiveness, high oxidation resistance, and low-temperature decomposition without the need for vacuum or inert gas atmospheres [25,26]. Cu salts (formate, acetate, and oleate) were used as Cu precursors, combined with amine compounds (Cu-complex), and fused with various solvents to prepare Cu-complex inks. After deposition on polymeric substrates, Cu-complex inks were self-reduced to metallic Cu below 140 °C, which was comparable to the glass transition temperature of polymeric substrates, demonstrating their benefits for flexible electronics fabrication. Moreover, the size, shape, and density of Cu nanoparticles can be systemically controlled by varying the concentration of amine compounds [27]. Recently, Cu-complex ink reduction and sintering were performed under ambient conditions following a rapid heating process within milliseconds or microseconds via a photo-thermal reaction [28].

The photo-thermal reaction is a well-known phenomenon where heat is generated by irradiation with high-intensity lights, such as xenon flash lamps, microwaves, and lasers, due to the localized surface plasmon resonance of electrically conductive nanomaterials [29,30,31]. This process enables thermal heating of materials and stimulates a range of physical processes (e.g., welding of metal nanowires and sintering of metal particles) and chemical reactions (e.g., synthesis of metal nanoparticles) [32,33,34]. Due to the ultrafast and selective heating at the surface of electrically conductive nanomaterials, this process offers the advantage of rapid treatment of heat-sensitive polymeric substrates without deformation.

In this study, we introduced laser-activated single-walled CNTs (SWCNTs) as flexible printed electrodes. Among the various promising materials for next-generation flexible electronics, SWCNTs have been studied for their high electrical conductivity and flexibility due to the sp^2^ hybrid conjugated bonding in their carbon structures. By using SWCNTs and a Cu-complex dispersion (SWCNT/Cu-complex), we fabricated flexible electrodes composed of SWCNTs and Cu (SWCNT/Cu) hybrid materials. This was achieved through the photo-thermally activated reductive synthesis of Cu nanoparticles with sintered morphologies induced by the π-plasmon resonance of SWCNTs. The π-plasmons of SWCNTs interact strongly with ultraviolet–visible (UV–vis) light, leading to spontaneous photo-thermal conversion. We confirmed that rapid synthesis of Cu nanoparticles on SWCNTs could be achieved under ambient conditions in air. The surface morphologies and electrical properties of the SWCNT/Cu hybrid electrodes were systematically controlled by varying the SWCNT concentration and laser output power.

## 2. Materials and Methods

### 2.1. Materials and Preparation of SWCNT/Cu-Complex

SWCNT powder was purchased from OCSiAl (TUBALL, purity > 93%, Leudelange, Luxembourg). Cu(II) formate tetrahydrate and 2-ethyl-1-hexylamine were purchased from Merck (Darmstadt, Germany) and used without further purification. Copper (II) formate tetrahydrate (1 equiv.) powder was added to 2-ethyl-1-hexylamine (4 equiv.) and stirred for 10 h to form a Cu-complex solution, as reported previously [35]. After the powder was completely dissolved, varying concentrations of SWCNTs (0.1, 0.5, 1.0, and 2.0 wt%) were added to the Cu-complex solution, and the mixture was stirred with a magnetic stirring bar for 24 h and an overhead stirrer for 24 h.

### 2.2. Fabrication and Characterization of the SWCNTs/Cu Electrodes

The SWCNT/Cu films were prepared by bar coating the SWCNT/Cu-complex solution onto glass and PET substrates. Bar coating was performed using a thickness-adjustable applicator with a thickness of 50 µm. The coated SWCNT/Cu films were irradiated using a laser (455 nm, xTool D1 Pro, xTool) at a power of 1–8 W and an operating speed of 8 mm/s. For comparison, the Cu-complex-based films were heat-treated in a conventional tube furnace at 175 °C with a ramping rate of 3.75 °C·min^−1^ in an Ar atmosphere for 1 h. X-ray diffraction (XRD) data were collected using an X’Pert PRO MPD instrument (PANalytical, Malvern, UK). Temperature profiles were measured using an infrared (IR) camera (RSE30, FLUKE, Everett, WA, USA) with the help of software (Smartview IR ver. 1.0.3.144, FLUKE, Everett, USA) by FLUKE. Thermogravimetry (TG) and differential scanning calorimetry (DSC) were performed using a Q600 instrument (TA Instruments, Waters, New Castle, DE, USA). The line pattern of SWNCT/Cu was observed using an optical microscope (OM; ECLIPSE LV100, Nikon, Minato, Japan). A direct current power supply (OPS-303, ODA TECHNOLOGIES, Incheon, Republic of Korea) was used to conduct light-emitting diode (LED) lighting tests and to evaluate the Joule heating performance of the fabricated SWCNT/Cu electrodes. Heating images were captured using an IR camera. Bending tests were performed using a source meter (2636 B, Keithley, Cleveland, OH, USA) connected to the current-measurement bending equipment. Structural morphologies were examined using scanning electron microscopy (SEM; S-4800, Hitachi, Chiyoda, Japan).

## 3. Results and Discussion

**Figure 1:**
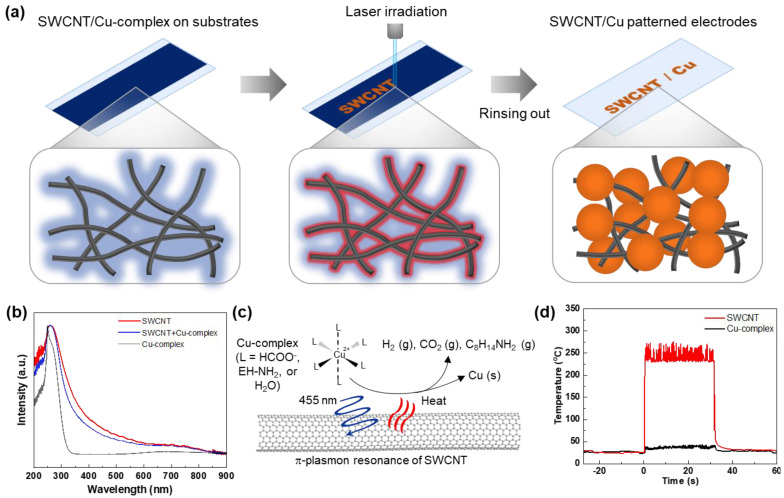
Laser-induced photo-thermal reaction for ultrafast preparation of the SWCNTs/Cu hybrid electrodes. (**a**) Schematic of fabrication process of patterned SWCNT/Cu electrodes. (**b**) UV–vis absorption spectra of Cu-complex, SWCNT/Cu-complex, and SWCNT. (**c**) Demonstration of photo-thermal and chemical reactions of SWCNT/Cu-complex producing metallic Cu. (**d**) Temperature profiles of SWCNT and Cu-complex when irradiated using laser at 455 nm.

As illustrated in Figure 1a, patterned flexible SWCNT/Cu electrodes are prepared by laser-induced photo-thermal reaction. The SWCNT/Cu-complex solution was coated onto the glass or PET film by bar coating. The SWCNT/Cu-complex films were irradiated at a laser wavelength of 455 nm to produce metallic Cu particles hybridized with SWCNTs. After rinsing the nonirradiated SWCNT/Cu-complex with ethanol, patterned flexible hybrid electrodes were obtained. This simple laser-induced patterning process originates from the light absorption of SWCNTs (Figure 1b) near 455 nm owing to the π-plasmon of the SWCNTs, resulting in nonradiative relaxation and photo-thermal conversion. Heat was generated at the surface of SWCNTs, and the temperature spontaneously increased within a few milliseconds. Thermal energy is then transferred to the neighboring Cu-complex and used as a thermal source for the decomposition of Cu-complex (Figure 1c). Time-resolved temperature distribution for a fixed exposure time of 32 s is measured to verify the effect of SWCNTs on laser-induced photo-thermal conversion (Figure 1d). The temperature profile for SWCNTs exhibited a rapid temperature increase of up to 200 °C within 0.5 s owing to the direct conversion of absorbed light into heat. This rapid temperature increase facilitated the thermal reduction of the Cu-complex and subsequent synthesis of metallic Cu nanoparticles. However, only the Cu-complex sample exhibited a negligible increase in temperature. This finding was attributed to the absence of the absorption behavior of Cu-complex with 455 nm light, even though a high absorption intensity was observed in the range of 250–300 nm.

**Figure 2:**
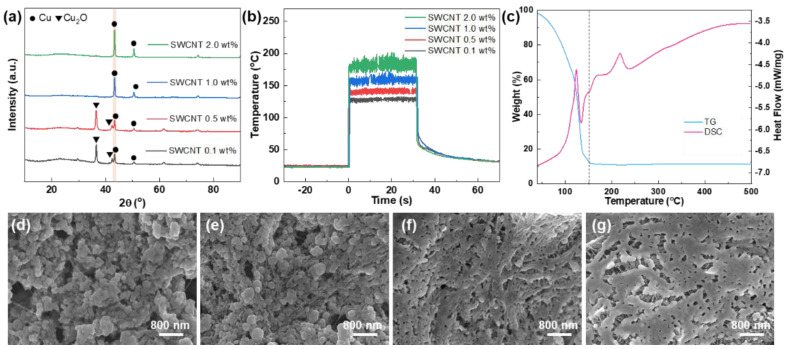
SWCNT concentration dependence on photo-thermal reaction. (**a**) X-ray diffraction patterns of SWCNT/Cu electrodes and (**b**) temperature profiles during laser irradiation of SWCNT/Cu-complex films at different SWCNT concentrations on glass substrates. (**c**) Thermogravimetry and differential scanning calorimetry curves of Cu-complex at a heating rate of 5 °C min^−1^ under air flow of 100 mL min^−1^. Scanning electron microscopy (SEM) images of SWCNT/Cu electrodes at SWCNT concentrations of (**d**) 0.1 wt%, (**e**) 0.5 wt%, (**f**) 1.0 wt%, and (**g**) 2.0 wt%.

To examine the reduction of Cu nanoparticles via the photo-thermal conversion of SWCNTs, laser irradiation was conducted on SWCNT/Cu-complex films deposited on glass substrates at varying SWCNT concentrations of 0.1, 0.5., 1.0, and 2.0 wt% at a laser power of 4 W for 30 s. As shown in XRD patterns (Figure 2a), the primary peaks of Cu (s) for SWCNT concentrations of 0.1 and 0.5 wt% are observed at 2 theta values of 43.3° and 36.5°, which correspond to the (111) crystal plane of metallic Cu and the (110) plane of Cu_2_O, respectively. However, under the experimental condition of 1.0 wt%, the Cu_2_O peak nearly vanished, and under the condition of 2.0 wt%, the result exhibited only metallic Cu (s) peaks. This finding indicated that as the SWCNT concentration increased, the reduction of Cu-complex to metallic Cu (s) occurred successfully because of the enhanced photo-thermal conversion efficiency via the π-plasmon density increase in SWCNTs. Figure 2b shows the temperature profiles of the SWCNT/Cu-complex films measured during laser irradiation. The average temperatures of 1.0 and 2.0 wt% samples were 160 and 185 °C, respectively, indicating that the temperature was much higher than the critical reduction temperature point of the Cu-complex. To further examine the relationship between the temperature and the reduction of Cu (s), TG and DSC analyses are performed (Figure 2c). Upon increasing the temperature up to 100 °C, the weight of the Cu-complex gradually decreased, indicating solvent evaporation. Significant weight loss occurred at temperatures in the range of 120–150 °C because of ligand dissociation of the Cu-complex. Based on the decomposition mechanism of Cu-complexes investigated by several groups, Cu-complexes may follow the next steps to form metallic Cu (s) (Equations (1)–(3)) [26,36].

Cu(HCOO)_2_L_4_ → Cu(HCOOH)L_2_ + 2L (1)

Cu(HCOO)L_2_ → Cu(HCOOH) + CO_2_ (g) + 1/2H_2_ (g) + 2L (2)

Cu(HCOO) → Cu (s) + CO_2_ (g) + 1/2H_2_ (g) (3)

This finding was reflected by the presence of several endothermic and exothermic peaks in the DSC curve. The conversion of Cu(II)-complex to Cu(0) took place through several steps, and metallic Cu (s) was successfully prepared over 150 °C. Accordingly, metallic Cu XRD peaks are shown for samples with SWCNT concentrations of 1.0 and 2.0 wt%. To verify this, the effect of SWCNT concentration on the thickness and electrical properties of the hybrid electrodes was investigated (Figure S1). The electrical resistivity decreased with increasing the SWCNT concentration up to 1 wt%, as Cu-complex could be efficiently reduced to metallic Cu (s) due to the enhanced photo-thermal conversion efficiency via the π-plasmon density increase in SWCNTs at high concentrations. The electrical resistivity reached a minimum value of 725 ohm·cm at 1.0 wt% and increased slightly at higher concentrations due to reduced relative Cu contents. The morphologies of the SWCNT/Cu hybrid materials were determined using SEM, and it is observed that Cu nanoparticles were synthesized and decorated on well-dispersed SWCNTs. The surface morphologies of samples using 0.1 and 0.5 wt% SWCNTs exhibited surface defects in the form of small prominences, indicating Cu oxide and resulting in particle inhomogeneity (Figure 2d,e) [37,38]. The formation of small Cu oxide prominences resulted from an insufficient reduction reaction due to the low photo-thermal conversion efficiency. These oxide prominences were completely diminished at higher SWCNT concentrations of 1.0 and 2.0 wt% (Figure 2f,g). Notably, Cu nanoparticles were sintered in the same shape and direction as the SWCNTs, leading to the superior electrical and mechanical properties of the SWCNT/Cu hybrid electrodes.

**Figure 3:**
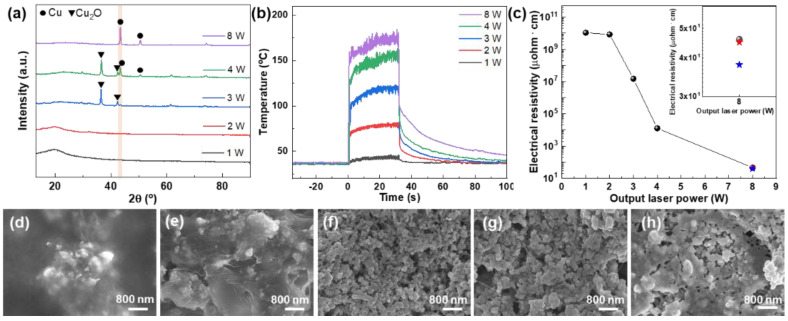
Laser power dependence on photo-thermal reaction. (**a**) X-ray diffraction patterns of 0.1 wt% SWCNT/Cu electrodes with respect to laser irradiation power, (**b**) temperature profiles during laser irradiation, and (**c**) electrical resistivity of SWCNT/Cu electrodes fabricated with various laser powers on a glass substrate. Electrical resistivities of thermally treated samples of Cu-complex (blue star) and 0.1 wt% SWCNT/Cu (red star) in the furnace are shown in the inset. (**d**–**h**) SEM images of SWCNT/Cu electrodes irradiated with output laser powers of 1, 2, 3, 4, and 8 W, respectively.

To further study the generation of Cu (s) via the photo-thermal reaction of SWCNTs, laser irradiation experiments are conducted at different laser output powers (Figure 3). The reduction of Cu-complex to Cu (s) is also confirmed, along with the increasing output power of the laser, by comparing the primary peak positions of Cu_2_O and Cu (Figure 3a). Under low irradiation conditions of 1 and 2 W, no specific metallic or oxide Cu peaks were observed. However, broad peaks at approximately 20° were observed. These peaks could be ascribed to the absorbed or intercalated molecules on the SWCNTs [39], indicating insufficient thermal energy to produce Cu-based particles from the Cu-complex. This finding is confirmed by the complete coverage of the SWCNTs with the Cu-complex, as shown in the SEM images in Figure 3d,e. Under 3 W laser conditions, the peaks assigned to Cu_2_O were observed at 36.5° and 42.3°. The saturated temperature of the 3 W laser is approximately 120 °C, which is slightly lower than the reduction and formation point of the metallic Cu nanoparticles, which is in agreement with the temperature profile shown in Figure 3b. Along with the oxide peaks, metallic Cu peaks at 43.3° and 50.5° are observed under the experimental conditions of 4 W laser irradiation, which is confirmed by the SEM images of metallic Cu particles with small Cu oxide prominences, as shown in Figure 3f,g. Only sharp metallic Cu peaks were observed for the sample at 8 W, in which the observed temperature of 175 °C was much higher than that during the reduction of Cu-complex. These results are in accordance with the photo-thermal conversion of SWCNT through π-plasmon resonance, as shown in Figure 2. Figure 3c shows the electrical resistivities of the SWCNT/Cu electrodes. As anticipated, the electrical resistivities of the SWCNTs/Cu electrodes decrease with increasing laser power at 8 W, exhibiting a minimum value of 46 μohm·cm. This value was comparable to that of the thermally treated samples in the furnace at 175 °C for 1 h under Ar atmosphere, as indicated by the blue and red stars (inset of Figure 3c). Additionally, the electrical resistivities of the SWCNT/Cu electrodes prepared under laser powers of 12 and 16 W were measured as 46.8 and 47.2 μohm·cm, respectively. These values are quite similar to the electrical resistivity of 46.0 μohm·cm for the 8 W sample, indicating that the Cu-complex is completely reduced at laser irradiation conditions over 8 W. We also confirmed that the optimized laser power through the thicknesses shows similar results (Figure S2). The high electrical property originated not only from the complete reduction of the Cu-complex to metallic Cu but also from the efficient coalescence of Cu nanoparticles by the effective photo-thermal reaction of SWCNTs, which is confirmed by the SEM image in Figure 3h. Therefore, we suggest that the laser-induced photo-thermal reaction is a highly effective and ultrafast method for producing metallic Cu nanoparticles and electrically conductive electrodes combined with SWCNTs under ambient conditions.

**Figure 4:**
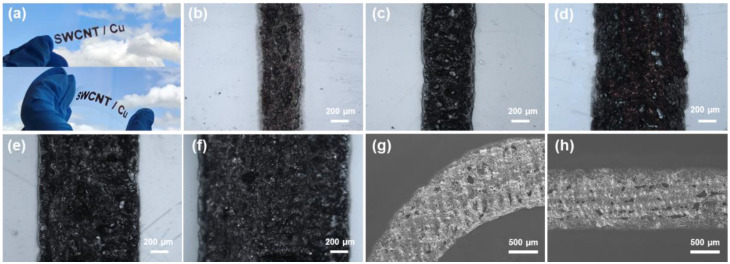
Laser-induced photo-thermal reaction for pattering. (**a**) Photographs of SWCNT/Cu patterned electrodes on PET substrate. (**b**–**f**) OM images of line patterns at different magnifications. (**g**,**h**) SEM images of curved and straight lines.

To fabricate the patterned electrodes, we performed a laser-induced photo-thermal reaction on the SWCNT/Cu-complex coated on PET substrates (Figure 4). Notably, laser-induced photo-thermal reactions are useful for patterning without any patterning masks [40,41], although ultrafine pattern morphologies, such as width and interpattern spacing, should be optimized with precise control of the wavelength, power, operating speed, and spot size of the laser. Figure 4a shows the patterning of SWCNT/Cu electrodes fabricated using 0.5 wt% SWCNT/Cu-complex under an 8 W laser-induced photo-thermal reaction. The widths of the line patterns are controlled at 500, 600, 1000, 1200, and 1600 µm, although the line edges are not perfectly clean (Figure 4b–f). SEM images show that a curved line is well established without severe defects, similar to a straight line (Figure 4g,h). This result indicates that the laser-induced photo-thermal reaction is useful for the direct patterning of flexible electrode applications for the SWCNT/Cu hybrid materials.

**Figure 5:**
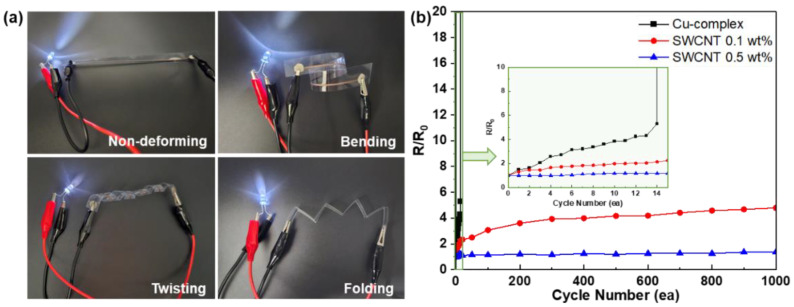
Flexibility of the SWCNT/Cu electrodes. (**a**) Photographs of flexible SWCNT/Cu electrodes under various deformation states. (**b**) Repeated bending test results of the Cu and SWCNT/Cu electrodes with 0.1 and 0.5 wt% SWCNTs. Inset shows the change in electrical resistance of the samples during low bending cycles.

In addition, we examined the flexibility of patterned electrodes under deformation (Figure 5a). Under various deformation states, such as 360° bending, twisting, and even 180° folding, the electrical properties of the SWCNT/Cu electrodes were maintained, as demonstrated by LED lightning. This finding was attributed to the well-dispersed, highly flexible SWCNT networks hybridized with highly conductive Cu nanostructures, as previously observed in the SEM images. For further evaluation of high durability, long-term bending tests for the SWCNT/Cu electrodes with 0.1 and 0.5 wt% SWCNTs were performed (Figure 5b). For comparison, Cu electrodes were fabricated by thermal treatment of the Cu-complex without SWCNT hybridization. For the Cu electrodes, the resistance change (R/R_0_) dramatically increased to 5455 even after 15 bending cycles under deformation conditions with a bending radius of 5R, whereas 0.1 wt% SWCNTs with Cu-complex samples retained an R/R_0_ value of 4.467 at 1000 bending cycles. Moreover, 0.5 wt% SWCNTs in the Cu-complex sample exhibited extremely high durability, with an R/R_0_ value of 1.281 after 1000 bending cycles. These results indicated that SWCNTs can act as efficient electrical and mechanical supports for hybrid materials under severely deformed conditions.

**Figure 6:**
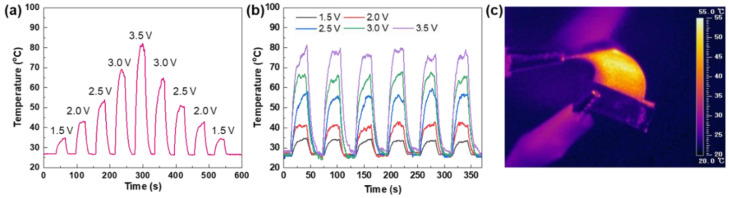
Joule heating performances of electrodes. (**a**) Temperature profile of stepwise increase and decrease in applied voltage ranging from 1.5 to 3.5 V, (**b**) temperature profile at applied voltage ranging from 1.5 to 3.5 V with repeated cycling, and (**c**) infrared camera image of SWCNT/Cu electrodes in bending state.

The SWCNT/Cu electrodes on the PET substrates can also be used as electrothermal film heaters, as shown in Figure 6. With increasing voltage from 1.5 to 3.5 V with a step size of 0.5 V, the SWCNT/Cu electrodes demonstrated a fast temperature increase, as shown in Figure 6a, indicating that the electrode temperature is readily adjusted by controlling the voltage. At an applied voltage of 3.5 V, the electrode temperature reaches 80 °C within 17 s, and this maximum value remains unchanged during repeated cycling, although a minute temperature fluctuation in the on-state is observed (Figure 6b). In addition, as shown in Figure 6c, a uniform temperature distribution of the electrode is observed in the IR camera image, indicating a negligible change in the electrode resistance and a well-interconnected SWCNT/Cu hybrid material with high flexibility. The Joule heating performances with high flexibility for the SWCNT/Cu electrodes are comparable to previous results for CNT/metal hybrid materials-based flexible electrodes [19,42].

## 4. Conclusions

In summary, laser-induced ultrafast reduction of Cu-complex has been successfully demonstrated to fabricate the SWCNT/Cu electrodes via photo-thermal conversion derived from the π-plasmon of SWCNTs. Through the laser irradiation, SWCNTs generate heat, which triggers the reaction of the Cu-complex to Cu at ambient conditions. The optimized laser irradiation condition yields sintering of Cu nanoparticles on SWCNTs and an electrical resistivity of 46 µohm·cm. Moreover, by coating SWCNT/Cu-complex solutions onto polymeric substrates and applying laser irradiation, we achieved efficient reduction of Cu-complexes to metallic Cu nanoparticles within a flexible SWCNT matrix. The results showed that increasing SWCNTs concentration improved the photo-thermal conversion and enhanced Cu reduction, resulting in electrodes with low electrical resistivity and high flexibility. The electrodes maintained excellent performance under bending, twisting, and folding deformation, demonstrating their potential for various applications. Additionally, the ability to pattern electrodes without masks and their use as electrothermal film heaters further highlight the practical advantages of this method. Overall, this approach offers a simple and effective way to produce flexible, conductive materials for advanced electronic applications.

## Data Availability

The data presented in this study are available on request from the corresponding author.

## Supplementary Materials

The following supporting information can be downloaded at: https://www.mdpi.com/article/10.3390/nano14171454/s1, Figure S1: Thickness and electrical resistivity of the SWCNTs/Cu electrodes fabricated with various SWCNT concentrations; Figure S2: Electrical resistivity as a function of the output laser power for the SWCNTs/Cu electrodes with a thickness of 12 µm and 21 µm. The sample was fabricated with 0.1 wt% SWCNT/Cu-complex.
