# Supplementary material for: Laser-Assisted Photo-Thermal Reaction for Ultrafast Synthesis of Single-Walled Carbon Nanotube/Copper Nanoparticles Hybrid Films as Flexible Electrodes
Source: Nanomaterials (Basel). 2024 Sep 6;14(17):1454. doi: 10.3390/nano14171454 (PMC11397256; doi:10.3390/nano14171454)
Supplement: Supplementary file 1 [file nanomaterials-14-01454-s001.zip › nanomaterials-3185860-supplementary.pdf]

Supporting Information

Laser-Assisted Photo-Thermal Reaction for Ultrafast Synthesis of Single-Walled Carbon Nanotube/Copper Nanoparticles
Hybrid Films as Flexible Electrodes

Mi-Jeong Kim [1,2] and Hee-Jin Jeong [1,2,*]

[1] Nano Hybrid Technology Research Center, Korea Electrotechnology Research Institute (KERI),
Changwon 51543, Republic of Korea; jeong0124v@gmail.com
[2] Department of Electro-Functionality Material Engineering, University of Science and Technology (UST), Changwon 51543, Republic of Korea
* Correspondence: wavicle11@keri.re.kr

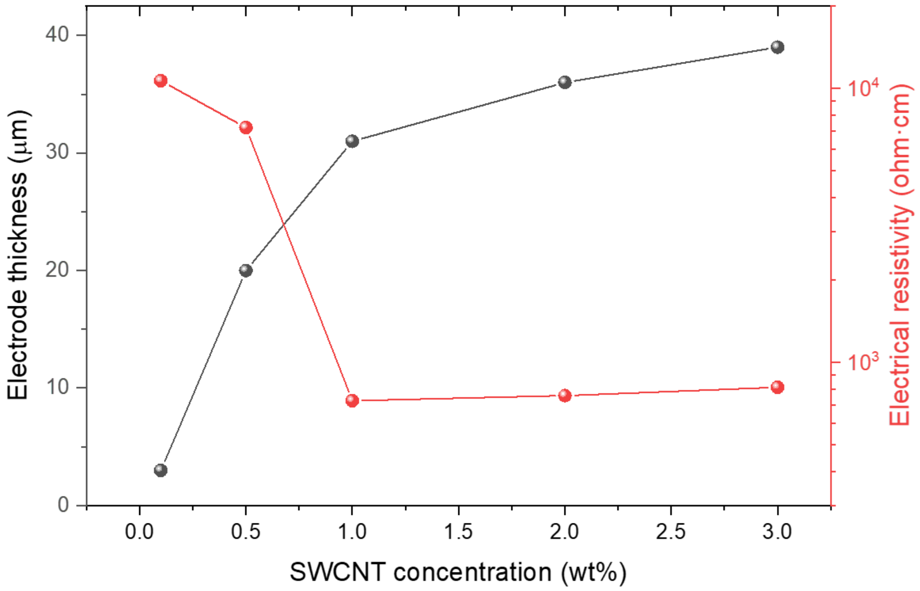

Figure S1. Thickness and electrical resistivity of the SWCNTs/Cu electrodes fabricated with various SWCNT concentrations.

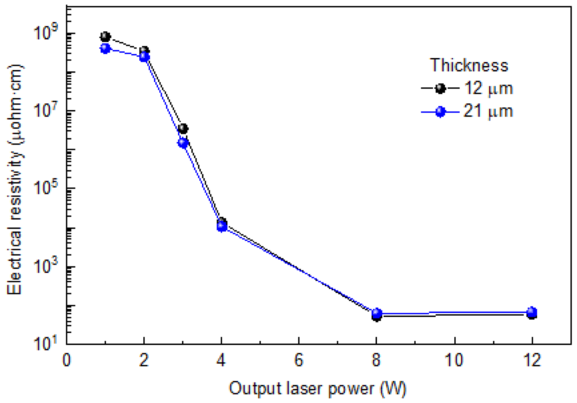

Figure S2. Electrical resistivity as a function of the output laser power for the SWCNTs/Cu electrodes with a thickness of 12 μm and 21 μm. The sample was fabricated with 0.1 wt% SWCNT/Cu-complex.
